# Interdigital Capacitor-Based Passive LC Resonant Sensor for Improved Moisture Sensing

**DOI:** 10.3390/s20216306

**Published:** 2020-11-05

**Authors:** Kristian Chavdarov Dimitrov, Sanghun Song, Hyungjun Chang, Taejun Lim, Yongshik Lee, Byung-Jae Kwak

**Affiliations:** 1 Department of Electrical and Electronic Engineering, Yonsei University, Seoul 03722, Korea; kristian.d@yonsei.ac.kr (K.C.D.); ssh5598@yonsei.ac.kr (S.S.); hyungjunchang@yonsei.ac.kr (H.C.); taejunim@yonsei.ac.kr (T.L.); 2 QUI Inc., Daejeon 34129, Korea; bjkwak@quiinc.com

**Keywords:** RFID, remote sensing, interdigital capacitor, fringing capacitance, sensor tag, moisture sensing

## Abstract

Herein, a passive low-profile moisture sensor design based on radio frequency identification (RFID) technology is proposed. The sensor consists of an LC resonant loop, and the sensing mechanism is based on the fringing electric field generated by the capacitor in the circuit. A standard planar inductor and a two-layer interdigital capacitor (IDC) with a significantly higher fringing capacitance compared to that of a conventional parallel plate capacitor (PPC) are used, resulting in improved frequency offset and sensitivity of the sensor. Furthermore, a sensor tag was designed to operate at an 8.2 MHz electronic article surveillance (EAS) frequency range and the corresponding simulation results were experimentally verified. The IDC- and PPC-based capacitor designs were comprehensively compared. The proposed IDC sensor exhibits enhanced sensitivity of 10% in terms of frequency offset that is maintained over time, increased detection distance of 5%, and more than 20% increase in the quality factor compared to sensors based on PPC. The sensor’s performance as a urine detector was experimentally qualified. Additionally, it was shown experimentally that the proposed sensor shows a faster response to moisture. Both simulation and experimental data are presented and elucidated herein.

## 1. Introduction

Moisture and water content sensing is a prerequisite for various industrial applications, such as urine sensors in the medical industry [1,2,3], sensing moisture content in concrete in the construction industry [4], and sensing soil moisture content in the agricultural industry [5]. The precise sensing of moisture content offers significant control over manufacturing processes, product yield, and quality assurance. Moreover, a sensor with non-contact remote sensing capability for these applications can be permanently embedded in the test substances or in places that are otherwise unreachable and hazardous.

In most applications, changes in the bulk conductivity [6,7] or dielectric properties [8,9,10] of the environment are measured to determine the moisture content of the medium under test. An increase in the moisture content increases the leakage current through the medium which can be measured. Therefore, conductivity measurement techniques require use of active sensors, rendering such techniques unsuitable for certain applications. For remote detection, additional active circuitry has to be embedded within the sensor, which complicates the design and increases the sensor cost. Moreover, water has a substantially high relative dielectric permittivity [11] ε_r_, typically in the range of 80–90. With an increase in the local moisture content, the effective dielectric constant also increases. This mechanism is utilized for moisture detection using a capacitor because its capacitance is a function of the dielectric constant of the material permeated by the electric field lines between its two electrodes. In a practical capacitor, all field lines are not contained in the region between the two parallel plates. The edge effect at the boundary of the capacitor generates fringing fields outside the parallel plate region. Prior studies have shown that these fringing fields are also functions of the dielectric constant of the surrounding medium [12]. Closed form solutions for a simple parallel plate capacitor (PPC) have been derived in which the approximate value of the capacitance is given by the expression:

(1) $$C\approx \epsilon_{r}\epsilon_{0}\frac{A}{d}+\frac{\varepsilon_{r}\varepsilon_{0}}{2\pi }\ln\left(\frac{2\pi A}{d}\right)\;\left[F\right],$$

where A is the area of the capacitor’s plates and d is the separation between them. The first and second terms on the right-hand side of the equation represent the capacitance of the parallel plate region and the fringing capacitance arising from the edge effect, respectively. The change in capacitance can be detected if the capacitor is included in an LC resonant circuit with resonant frequency f_0_ determined by the expression:

(2) $$f_{0}=\frac{1}{2\pi \sqrt{L\cdot C}}\;\left[\mathrm{Hz}\right].$$

If there is no magnetic material (i.e., with magnetic permeability μ_r_ ≠ 1) in proximity to the sensor, the resonant frequency of the loop solely depends solely on the capacitance, implying that f_0_ is a function of the effective permittivity of the surrounding medium.

**Figure 1:**
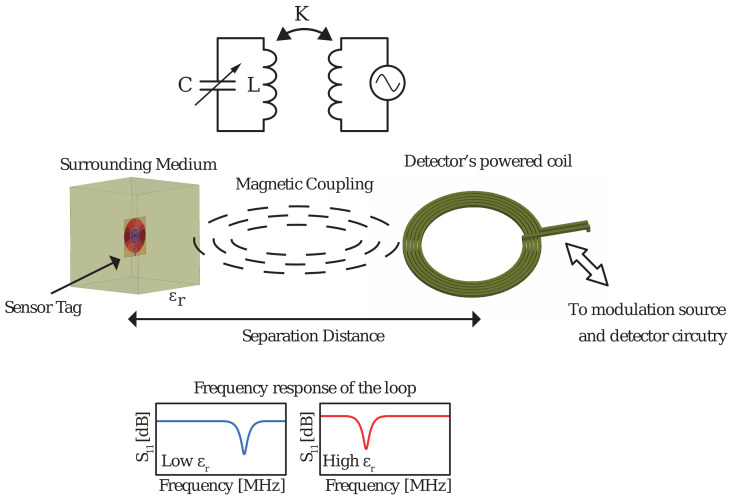
Operation principle of inductively coupled radio frequency identification (RFID) sensor tag with its equivalent circuit Examples of frequency responses for different permittivities of the surrounding medium are also shown values.

If the LC circuit is magnetically coupled at its resonant frequency f_0_ to a driving coil located in a detector [13], then the LC loop is practically short-circuited at resonance and draws power from the coil. This creates a notch response at the detector side that can be detected as shown in Figure 1.

In this paper, a moisture detection passive LC resonant sensor is presented. The proposed sensor exhibits improved sensing characteristics compared to those of similar passive sensors [14]. The sensor tag features an interdigital capacitor (IDC) as a sensing element [15,16,17]. Over a standard parallel plate capacitor, an IDC has the significant advantage of possessing considerably larger fringing fields, which are substantially affected by the surrounding medium. The sensor tags operational at an ultra-high frequency (UHF) of 8.2 MHz electronic article surveillance (EAS) [18] frequency band were designed and fabricated based on IDC and PPC. The sensors were designed to have a small area of less than 70 × 70 mm^2^, high maximized quality factor Q, and large frequency change when exposed to moisture. Experimental results reveal significant enhancement in the performance of the sensor based on IDC compared to that based on PPC 5% increase in the maximum detection distance and 10% increase in sensitivity based on frequency offset. Further, a faster response is possible due to its larger capacitor area, along with the stability in the sensitivity; a larger frequency offset is maintained over the same period of time.

## 2. Materials and Methods

### 2.1. Fringing Capacitance

**Figure 2:**
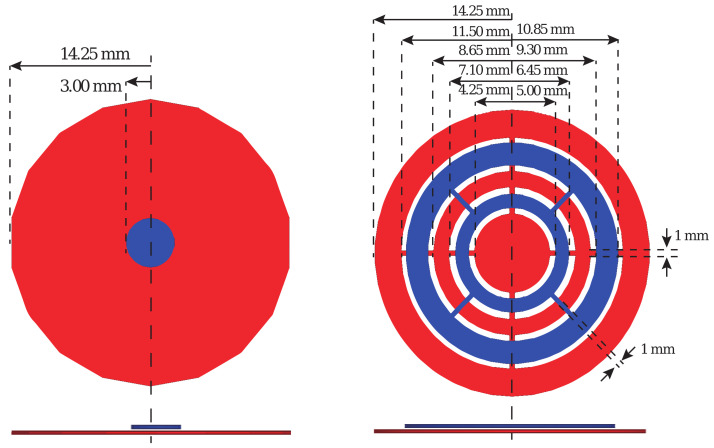
Sensing capacitors in this work with all relevant dimensions shown: on the left a standard parallel plate capacitor (PPC) with large bottom plate (in red) and smaller top (in blue) plate is shown; on the right, the proposed interdigital capacitor (IDC) based on an interdigital structure with two interconnected rings on the top layer (in blue), and two connected rings and a plate on the bottom layer (in red).

As discussed in Section 1 the sensing mechanism used for the detection is the change in capacitance of a planar capacitor, due to its fringing fields. This was achieved by implementing a two-layer interdigital structure. The proposed IDC consists of two annular rings on the top layer connected by four interconnects. Similarly, the bottom layer is made of two annular rings and a center plate connected in four locations. The top and bottom plates are non-overlapping, to minimize the fields contained between them and to maximize the generated fringing fields. This design was chosen over more standard IDC structures due to its improved fringing fields and shape, allowing capture of the additional capacitance between the inner-most winding of the inductors and the bottom plate of the capacitor, as described later in this work. The proposed design is compared to a standard PPC, which is shown in Figure 2. The large difference in size between the two PPC plates is to maximize the fringing fields and, as discussed later, to increase the interacting capacitance between the inductors, so that the frequency shift sensitivity due to moisture is maximized. When the two plates have the same size, the sensitivity will decrease because the area that can be exposed to moisture is limited and the generated fringing fields reduced. Both sense capacitor designs and relevant dimensions are shown on Figure 2.

The capacitances, resonant frequencies, and Q-factors of the sensor tag in surrounding media with dielectric permittivities of 1 and 10 are obtained. Permittivity of 1 corresponds to free space operation of the sensor and is used as a baseline for all performed measurements. Permittivity of 10 is the expected effective permittivity of the surrounding material in the wet state in the measurement setup in Section 3. The capacitors are chosen such that the resulting resonant frequency in air, after the inclusion of the inductor, is 8.2 MHz. The capacitance of the PPC is 18.3 and 20.5 pF for ε_r_ = 1 and ε_r_ = 10, respectively, indicating a capacitance difference ΔC of 2.2 pF between the two values. The same difference can also be expressed in terms of percentage, allowing relative comparison of different value capacitors:

(3) $$\Delta C=\left|\frac{C\left(\varepsilon_{r}=10\right)-C\left(\varepsilon_{r}=1\right)}{C\left(\varepsilon_{r}=1\right)}\right|\cdot 100\;\left[\%\right].$$

For comparison, the capacitance difference obtained by the proposed IDCs is 18.3 pF; notably, this value is substantially larger than those obtained by the design that employs a PPC. A summary of the obtained results is shown in Table 1.

Evidently, the capacitance of the PPC is primarily due to the field in the parallel-plate region, and only a small amount of the capacitance is due to the fringing field. The bounded field lines between the plates are unaffected by the surrounding medium, which limits the change in the capacitance. At the capacitor’s plate edges, a small portion of the fringing field is generated and extends outside the parallel plate region, which is the only field affected by the dielectric constant of the surrounding medium. In contrast, the proposed IDC has no overlapping section between the top and bottom layers, and its capacitance is mainly due to the fringing field, allowing the capacitor to exhibit large variation in capacitance. Consequently, a rapidly changing resonant frequency of the sensor tag is obtained. Usually, an IDC is constructed with a single layer [19]; however, in this case, a line across the inductor’s winding is required to close the resonant circuit. Therefore, a double-layer structure is employed. This expedites the design procedure because the capacitance can be controlled easily by the amount of overlap between the fingers on the two layers.

**Figure 3:**
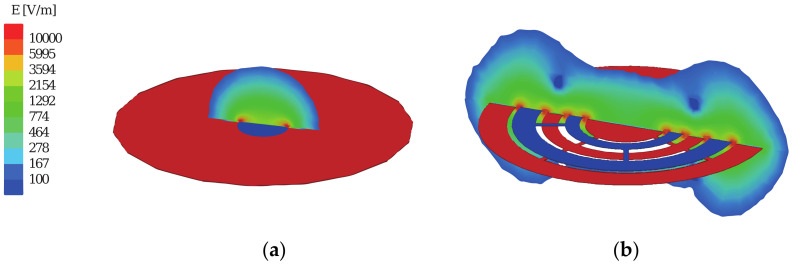
Fringing electric fields generated by the sensor tag using: (**a**) PPC; (**b**) IDC.

The proposed IDC does not have any overlapping section between the top and bottom plates; therefore, the capacitor needs a significantly larger physical area to achieve the same capacitance value as a PPC. Nevertheless, the larger area where the field exists offers a considerable advantage for the proposed moisture sensor. Additionally, the effective dielectric constant of the moisture content, which determines the capacitance change, is integrated over this large area, effectively reducing discrepancies in the detection, particularly when there is a substantial variation in the amount of moisture across the capacitor. Figure 3a,b shows the simulated electrostatic fields in a PPC and IDC.

The PPC consists of two plates, of which the top is smaller than the bottom to enhance the fringing field. The entire field is concentrated around the smaller top plate as shown in Figure 3a. Because the resulting field is highly asymmetric, the sensor capacitor reacts to moisture differently depending on whether it is above the top plate or below the bottom plate. However, when an IDC is used (Figure 3b), the resulting fields are symmetric on both sides of the sensor capacitor; furthermore, because of the relatively small distance between the individual arms of the capacitor, the coupling between these arms and the inductor is similar irrespective of the position of the small/large capacitor plate.

### 2.2. Calculation of Capacitor Loss and Q-Factor

The quality factor Q of an LC tank circuit describes the resonance behavior and represents the loss of the resonator. A higher Q-factor leading to a sharper resonance and longer detection distances is generally desirable for the sensor tag. The Q-factor of a series RLC circuit is represented [20] as:

(4) $$Q=\frac{1}{\omega \mathrm{RC}}.$$

In Equation (4), ω = 2πf is the serial resonance frequency, C is the serial capacitance, and R is the serial resistance of the circuit. The resistance R is physically measured as the real part of the input resistance at the resonant frequency:

(5) R=ReZ1,1 at f=fres.

With the increase in the serial capacitance and serial resistance, the Q-factor of the circuit decreases; therefore, an IDC with a low capacitance value is employed initially. Inevitably, the capacitance increases under moisture exposure, and the Q-factor degrades, reducing the maximum detection distance of the sensor. Furthermore, increasing the number of turns in the inductor increases R; therefore, both C and L should be simultaneously maintained at low levels to maximize the Q value. However, for a low C, a large L is required to maintain f_max_. Because these two requirements are in contradiction, special attention must be paid to the choice of L and C to achieve a maximum Q-factor. When exposed to moisture, the capacitance of the IDC increases; moreover, it becomes the dominant term in determining the Q value. To maintain high Q, C needs to be low. In this case, L needs to be increased to maintain the same resonant frequency, which then decreases Q. Therefore, a compromise is required.

### 2.3. Sensor Design

**Figure 4:**
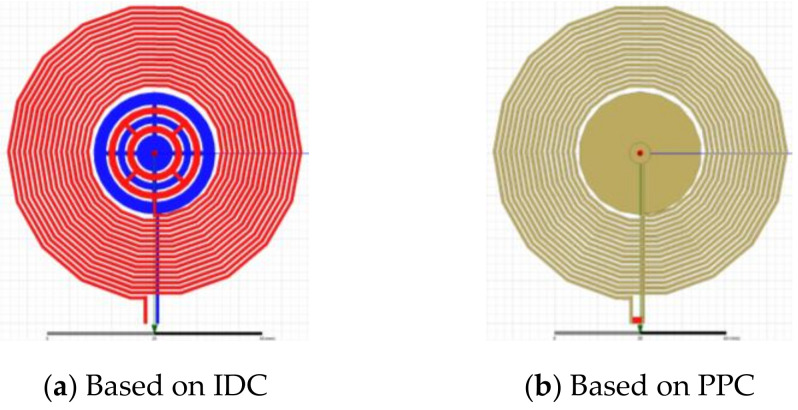
Simulation models of the two sensor tags: (**a**) sensor tag with proposed interdigital capacitor (**b**) sensor tag with conventional PPC having one small plate coplanar with the inductor and a larger plate located on the second layer below the small plate.

The static solver Maxwell^®^ by ANSYS was used to calculate the capacitance values of the designed sensors, PPC and IDC. Further, both capacitors were combined with the same inductor to calculate the resonant frequency ω of the LC tank circuit. To determine the ω quality factor Q of the loop, the real part of the input impedance R and the capacitance C are required. The two designs, shown in Figure 4, each consisting of the reference PPC and the proposed IDC, are compared.

The results are summarized in Table 2. Because the resonant frequency of the LC tank circuit is proportional to the square root of the capacitance value, frequency shifts of ~5.5% and ~30% are expected for the PPC and IDC designs, respectively. The absolute and relative frequency differences ΔF in Table 2 are calculated similarly to the values of ΔC in Table 1.

However, the interaction between the bottom plate of the capacitor and the inner-most windings of the inductor significantly contributes to the total capacitance of the sensor because these two components technically form a capacitor. Owing to the large area and gap between the two components, significant fringing fields exist between them; these fields increase the total capacitance by a factor of nearly 2, thereby dominating the change in the capacitance. Even after considering this effect, the IDC designs exhibit a superior sensitivity and Q-factor compared to the equivalent PPC design. Both sensors are expected to resonate at 8.2 MHz surrounded with a medium with a permittivity of 1, i.e., in free space. When switched to a surrounding medium of ε_r_ = 10, the resonant frequency of the IDC design reduces by 2.7 to 5.5 MHz, which is 7% more than that based on a PPD. Additionally, the IDC design retains its higher Q when the surrounding medium has permittivity of 10. This higher value is due to a lower real part of the sensor’s impedance. In the PPC design, most of the fields are contained within the lossy substrate increasing the loss, whereas in the case of the IDC, the field propagates though air due to its improved fringing fields. The higher Q of the IDC sensor implies that the maximum sensing distance will be longer than that of the PPC sensor with a lower Q.

### 2.4. Equivalent Circuit

**Figure 5:**
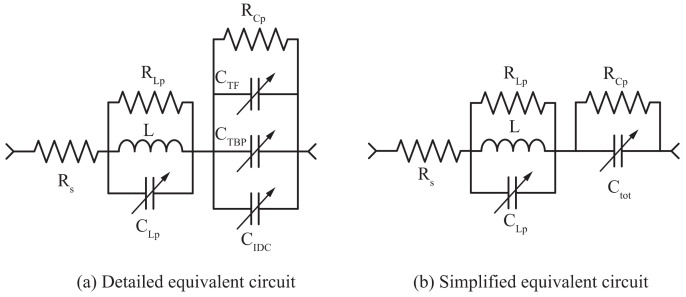
Equivalent circuit of RFID sensor tags: (**a**) detailed equivalent circuit including parasitic and loss elements; (**b**) simplified equivalent circuit used for the determination of the resonant frequency of the LC loop.

The serial resonance frequency of the sensor tag is determined by the inductance L of the coil and the capacitance of the IDC C_IDC_ in parallel with the parasitic capacitances associated with the inductor’s winding and feedline interconnection. The parasitic capacitance C_TBP_ is between the inner-most turns of the inductor coils and the capacitor plates located on opposite sides of the substrate, C_TF_ is between the windings and the shorting line, and C_Lp_ is the inductor’s turn-to-turn capacitance. These parasitic capacitances can be relatively large, and because they are in parallel connection with the main IDC, their effect on the serial resonance frequency of the tag may be significant. By increasing the distance between the inner-most windings of the coil and the capacitor plate, ***C_TBP_*** can be reduced. However, this increases the sensor dimensions; therefore, rather than minimizing ***C_TBP_***, the spacing can be optimized such that the interaction between the capacitor bottom plate and the coil produces significant fringing fields, which positively affect the tag’s frequency offset. Furthermore, the capacitance between the inductor and feed line C_TF_ cannot be significantly reduced and, therefore, its effects must be considered when designing the total serial capacitance. The equivalent circuit of the sensor is shown in Figure 5.

**Figure 6:**
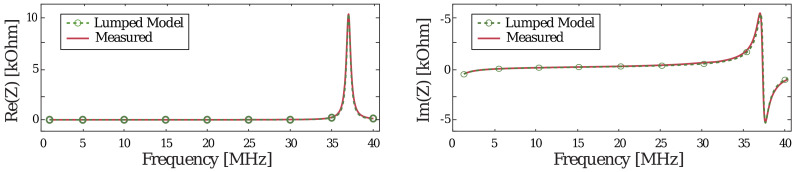
Measured and modelled input impedances.

The validity of the model was verified by comparing the directly measured input impedance of the fabricated sensors with the extracted input impedance via magneto- and electro-static simulations using ANSYS^®^ Maxwell. The most important regions of the extracted data are around the serial resonance of 8.2 MHz. In this region, the inductance and capacitance values mostly dominate over the parasitic components, because they determine the zero-crossing point in the imaginary impedance of the circuit. Another dominating factor is the serial resistance R_s_, which determines the real part of the impedance at low frequencies and is directly related to the quality factor of the resonator. The two resistances connected in parallel to the inductor, R_Lp_ and R_Cp_, and the capacitor account for the leakage, which determines the accuracy of impedance near the resonator’s parallel resonance at 36 MHz; therefore, they can be removed to simplify the equivalent circuit. As illustrated in Figure 6, the used model accurately approximates the measured data, and thus can be used to improve the design.

## 3. Results

**Figure 7:**
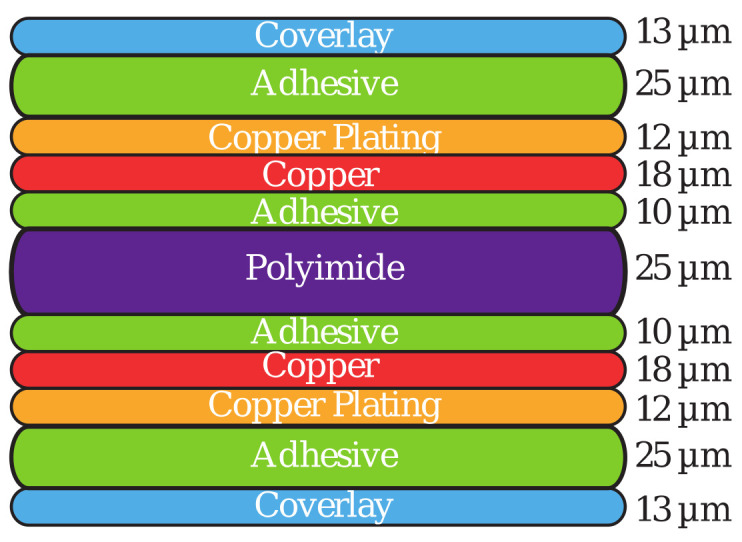
Stack-up used during fabrication. Additional top and bottom polyamide layers were added for sensor protection and isolation.

The RFID tag was fabricated via standard manufacturing processes used for flexible printed circuit boards (PCB). The base substrate was Dupont^®^ Kapton, a polyimide-based material, with a thickness of 25 μm. The sensor tag has a two-layer structure, as shown in Figure 7.

**Figure 8:**
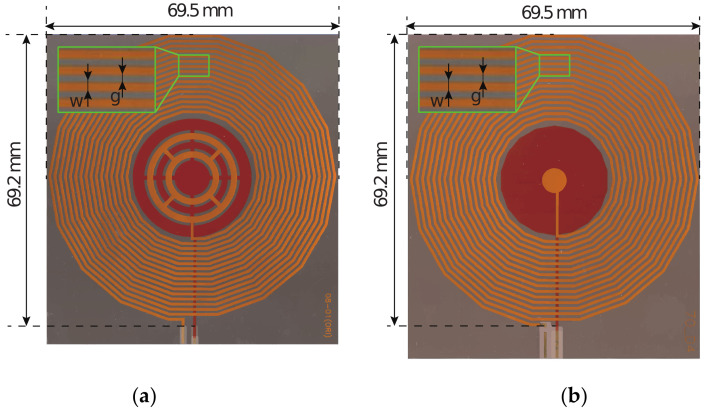
Manufactured sensor tags: (**a**) top and bottom views of the proposed IDC; (**b**) top and bottom views of the PPC.

The proposed RFID sensor tags consisted of 17-turn inductor coils with a trace width w = 0.70 mm and spacing g = 0.52 mm between the windings. The two sensors employing IDC and PPC are shown in Figure 2. The inductor and one of the sensing capacitor plates are located in one of the layers. In the other layer, the second capacitor plate and the interconnect line were located. The resonant circuit is closed by shorting the two layers using two via holes. In addition, to protect the copper layers from oxidation, the top and bottom of the tag assembly are covered with polyimide, which also provides additional mechanical strength. All layers are bonded using an adhesive material, resulting in a combined total thickness of 181 μm. The sensor tags were designed to fit in an area of 70 × 70 mm; however, an additional 5 mm was added to one side of the sensor tag to incorporate measurement terminals, as shown in Figure 8. Two via holes with a 0.4 mm radius are used to pass the bottom terminal of the capacitor to the other side. These terminals are used to connect a vector network analyzer (VNA) via super miniature type-A connectors (SMA) to the sensor tag to directly monitor the resonant frequency and input impedance of the sensor tag.

**Figure 9:**
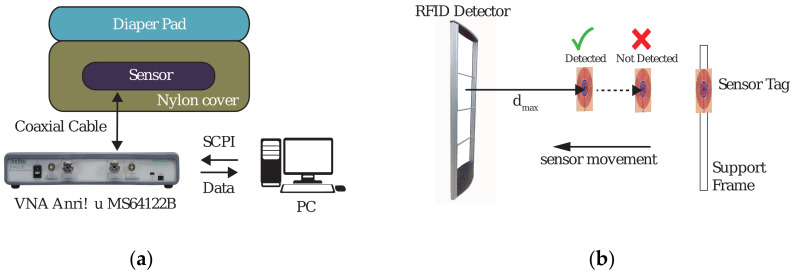
Measurement setups: (**a**) measurement setup for resonant frequency and frequency response to saline solution using a vector network analyzer (VNA); (**b**) measurement setup for maximum detectable distance Blozi RFID detector.

To quantify the performance of the sensor tag that employs the IDC, several practical tests and control experiments, such as detection distance measurement and determination of tag sensitivity to surrounding moisture, were performed. For moisture content, saline solution was used to wet commercial diapers. The measurement equipment and setups are shown in Figure 9, and the obtained results are discussed.

**Figure 10:**
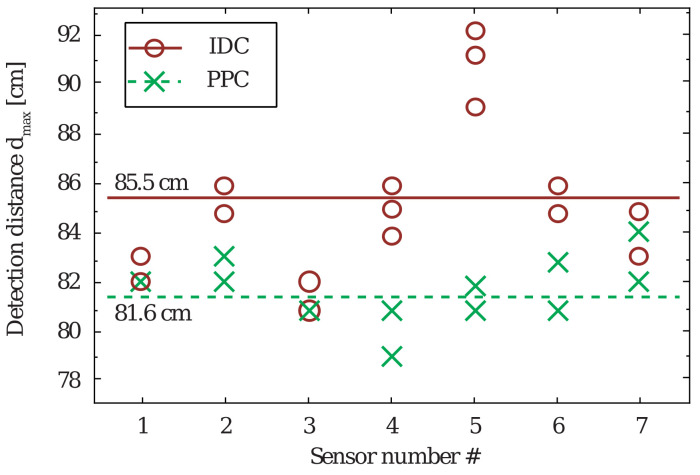
Measured maximum detection distance.

The distance detection measurements were performed using an off-the-shelf RFID tag detector, Blozi^®^, with a detection window between 7.8 and 8.7 MHz. All measurements were performed with constant detector sensitivity and constant sensor height. The detection distance was directly proportional to the loss in the sensor and its quality Q-factor. In the experiment, the detection distance d_max_ was recorded by attaching the sensor to a support frame and moving it toward the detector until detection occurred. The measurement was performed using seven different sensors of each type, and each measurement was repeated three times. The obtained data are plotted in Figure 10, in which the average values are shown with the solid and dashed lines for the IDC- and PPC-based sensors, respectively.

The measured results exhibit good consistency. The small difference in the maximum detection distance between the two types of sensors can be attributed to the manufacturing tolerances and moisture absorption by the polyamide substrate used in the construction [21]. In Figure 10, the measured detection distance for sensor #5 with an IDC is an outlier with an approximately 10% higher value than the average detection distance. Moreover, the measurement has a mean error of ±1 cm and a measurement resolution of 1 cm. The average maximum detection distances for the PPC-based and IDC-based sensors are 81.6 and 85.5 cm, respectively. Evidently, the detection distance is directly proportional to the quality factor of the sensor tag. The major sources of loss are the electric field in the capacitor of the resonant circuit and the ohmic loss in the inductor windings. Because the same inductors are used for all sensors, the difference in the maximum detection distance resulted from the increased free space Q-factor of the IDC-based sensor tag due to the enhanced fringing fields in the IDC design.

**Figure 11:**
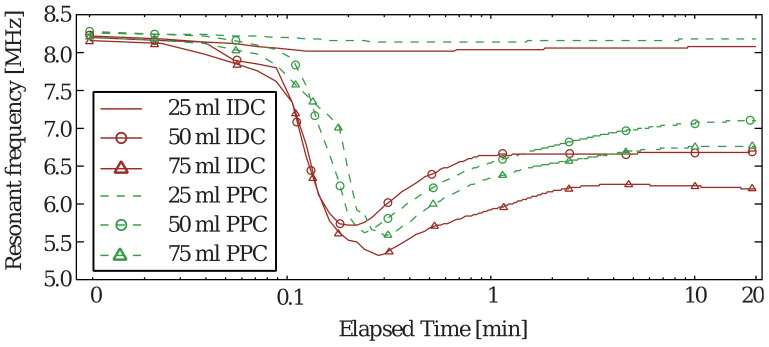
Typical transient response of the sensor when exposed to different amounts of saline solution.

In the second experiment, the sensor tag was assumed to be a urine detector. The setup is shown in Figure 9a. A diaper pad of the same size as the sensor was placed on top of the sensor. To protect the sensor and the measurement equipment from the liquid, the sensor was placed inside a nylon bag. Different amounts of saline solution were then added to the diaper pad. Subsequent changes in the resonant frequency of the sensor were measured using an Anritsu^®^ MS46122B VNA. This experiment was performed to quantify the frequency response and sensitivity of the sensors. Similar to the previous experiment, the frequency shift measurement was performed using five sensors of each type and repeated three times. In three separate experiments, 25, 50, and 75 mL of saline solution was spread over the entire area of the absorbing material while the resonant frequency was being logged. The frequency responses of the tags were recorded for 20 min with a sampling rate of 2 s. Typical time-response curves for the two types of sensors and different amounts of saline solution are shown in Figure 11. Initially, after applying the saline solution, the resonant frequency drops sharply to the lowest value within 10 s as the liquid settles in the diaper pad.

**Figure 12:**
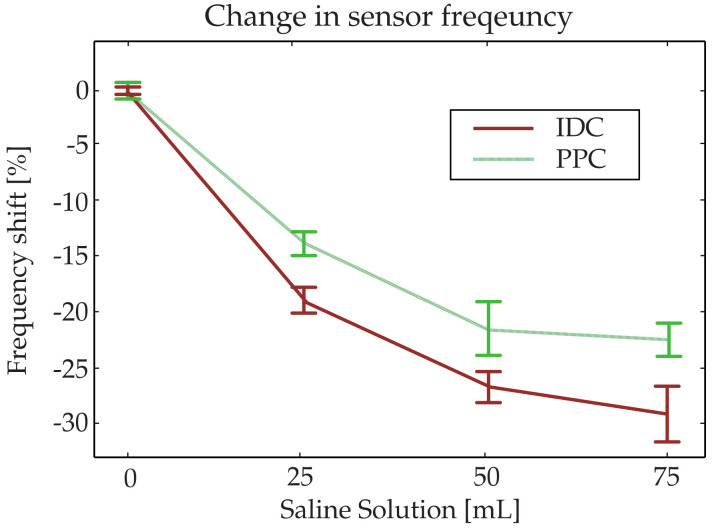
Steady state frequency shift of the two types of sensors when exposed to saline solution in a diaper with SE error bars.

A resonant-frequency recovery time of a few minutes is observed after, following which the resonant frequency of the sensor remains constant over time. First, the IDC-based sensor exhibits a lower peak resonant frequency and lower recovery frequency than that of the equivalent PPC sensor. This enhanced sensitivity is a great advantage because the detection error can be lowered significantly. Furthermore, the response is faster for the IDC-based sensor, which is also another advantage. In rare cases, such as in the experiment with 50 mL of saline solution, the PPC-based sensor achieves a lower absolute minimum frequency than that of the IDC-based sensor. However, after 1 min, the responses cross over, and the IDC-based sensor attains a lower recovery frequency. The variations in the frequency shift for the two types of sensors at the end of the 20-min recording time, as the recovery frequencies attain constant values, are shown in Figure 12. Regardless of the amount of saline solution, the proposed tag shows a larger frequency shift, again implying the enhanced sensitivity. As evidenced, the proposed sensor-based IDC shows a faster initial response regardless of the amount of saline solution, and the resonant frequency reaches the minimum before the PPC counterpart. Furthermore, the proposed tag recovers the resonant frequency slower than that based on PPC.

The quality factor Q of the fabricated sensors can be calculated based on the measured impedance parameters, resonant frequency, and capacitance value obtained from the equivalent model. The results are compared in Table 3. The measured Q-factor of the IDC sensor has improved with a 29% Q-factor. The obtained Q-factor ratios between the PPC and IDC are similar for the simulated and measured results. However, there is large absolute value difference between them. The lower measured Q-factor can be contributed to fabrication tolerances, lower conductivity, and substrate moisture abortion.

Finally, the presented sensor can be compared with others recently proposed in the academic literature relating to urine sensors. Direct parameter comparison can be somewhat difficult to be perform due to the wild variance in sensor types, sensor size, target markets, testing methodology, etc. However, there are still undeniable advantages and disadvantages of each sensor implementation.

The current work and other recent moisture sensors are compared in Table 4. The current work and that of [3] have significant advantages over those of [1,2], because the former are a passive type. Because active circuitry is needed in [1,2], they can be significantly more expensive than low-cost passive sensors. The sensor in [3] is similar to the demonstrated sensor because they both rely on the change in the resonant frequency of the LC tanks. However, the sensor in [3] is limited to binary detection, i.e., to detect the minimum level of moisture, whereas the proposed sensor can be applied to measure the relative level of moisture in the surroundings based on the amount of frequency shift. Therefore, the proposed sensor is more versatile, and can be used for moisture detection in various environments, including concrete.

## 4. Discussion

A planar, RFID-based moisture sensor tag that employs an IDC design is proposed to improve the frequency offset and sensitivity of the sensor. The two-layer interdigital sensing capacitor exhibits larger frequency offset for the same amount of moisture, which translates to a higher sensitivity. The IDC has improved the quality factor of the tag and consequently increases the maximum detection distance. The proposed sensor also retains a higher Q-factor when exposed to moisture, despite its larger frequency offset. Furthermore, the symmetrical structure allows sensing on sides of the tag, which is difficult to achieve with a conventional PPC because the two plates are generally designed to have a large difference in their size to increase the fringing field and, therefore, the sensitivity. The larger size of the IDC enables the integration of the dielectric properties of the material over a larger area, ensuring improved tolerance to moisture variations and faster initial response to large increments in moisture. Experimental results reveal an increase of as much as 5% in the maximum detection distance, and more importantly, up to 10% higher sensitivity based on the frequency shift due to the same amount of moisture, compared with the sensor based on a PPC. The IDC sensor exhibits faster frequency response and lower overall resonant frequency. The performance of the equivalent PPC- and IDC-based sensors as a diaper urine sensor was exhaustively measured and qualified in this work. Moreover, time response data of the sensor, missing from other similar papers [1,2,3], revealed a pattern of a rapid decline in frequency followed by a recovery region. Verifying the potential of the proposed sensor in other moisture-sensing applications remains as future work.

## Figures and Tables

**Table 1:** Simulated capacitance values and capacitance change for different values of effective permittivity of the surrounding medium.

Design	Center Capacitor		
	C [pF] (ε_r_ = 1/10)	ΔC	
		[pF]	[%]
PPC	18.3/20.5	2.2	12
IDC	15.6/33.9	18.3	117

**Table 2:** Simulated capacitance values, frequency shifts, and quality factors of the two sensors based on PPC and IDC.

Design	Total Capacitance			Frequency Shift			Q	
	C [pF] (ε_r_ = 1/10)	ΔC		F [MHz] (ε_r_ = 1/10)	ΔF		ε_r_ = 1	ε_r_ = 10
		[pF]	[%]		[MHz]	[%]		
PPC	27.9/49.0	21.1	76	8.2/6.1	2.1	26	30.0	22.6
IDC	28.6/59.8	31.2	109	8.2/5.5	2.7	33	37.0	23.6

**Table 3:** Q-factor results comparison table.

Design	Q-Factor (In Free Space)	
	Simulated	Measured
PPC	30	21
IDC	37 (+23%)	27 (+29%)

**Table 4:** Comparison table of recent moisture sensors.

Work	Sensor Type by			Base Material	Advantages	Disadvantages
	Powered	Sense Location	Sense Mechanism			
[1]	Active	Local	Resistive	Fabric	Accuracy	Expensive Requires power
[2]	Active	Local	Capacitive/Resistive	Diaper Foil		
[3]	Passive	Remote (RF 13.56 MHz)	LC Resonance	Paper/Polyacrylate		Binary detection
[22]	Passive	Remote (RF 14.00 MHz)	LC Resonance	Polyamide	Low cost	Large size
[23]	Passive	Remote (RF 35.75 MHz)	LC Resonance	PCB		Low efficiency
[24]	Passive	Remote (RF 177.00 MHz)	LC Resonance	LTCC	Sensitivity	Versatility
This work	Passive	Remote (RF 8.20 MHz)	LC Resonance	Polyamide	Low cost	Versatility
